# Substituted Triazole-3,5-Diamine Compounds as Novel Human Topoisomerase III Beta Inhibitors

**DOI:** 10.3390/ijms26136193

**Published:** 2025-06-27

**Authors:** Yasir Mamun, Somaia Haque Chadni, Ramanjaneyulu Rayala, Hasham Shafi, Shomita Ferdous, Rudramani Pokhrel, Adel Nefzi, Prem Chapagain, Yuk-Ching Tse-Dinh

**Affiliations:** 1 Biochemistry PhD Program, Department of Chemistry and Biochemistry, Florida International University, Miami, FL 33199, USA; ymamu001@fiu.edu (Y.M.); schad008@fiu.edu (S.H.C.); sferd001@fiu.edu (S.F.); anefzi@fiu.edu (A.N.); 2 Center for Translational Science, Florida International University, 11350 SW Village Parkway, Post Saint Lucie, FL 34987, USA; rraya002@fiu.edu (R.R.); hshafi@fiu.edu (H.S.); 3 Department of Physics, Florida International University, Miami, FL 33199, USA; rpokhrel@arizona.edu; 4 Herbert Wertheim College of Medicine, Center for Translational Science, 11350 SW Village Parkway, Post Saint Lucie, FL 34987, USA; 5 Biomolecular Sciences Institute, Florida International University, Miami, FL 33199, USA

**Keywords:** topoisomerase, TOP3B, tyrosine kinase inhibitors

## Abstract

Human topoisomerase III beta (hTOP3B) is a unique and important enzyme in human cells that plays a role in maintaining genome stability, affecting cellular aging, and potentially impacting viral replication. Its dual activity on both DNA and RNA makes it a valuable target for therapeutic interventions. hTOP3B has been shown to be required for the efficient replication of certain positive-sense ssRNA viruses including Dengue. We performed in silico screening of a library comprising drugs that are FDA-approved or undergoing clinical trials as potential drugs to identify potential inhibitors of hTOP3B. The topoisomerase activity assay of the identified virtual hits showed that bemcentinib, a compound known to target the AXL receptor tyrosine kinase, can inhibit hTOP3B relaxation activity. This is the first small molecule shown to inhibit the complete catalytic cycle of hTOP3B for the potential interference of the function of hTOP3B in antiviral application. Additional small molecules that share the *N*^5^*,N*^3^-1*H*-1,2,4-triazole-3,5-diamine moiety of bemcentinib were synthesized and tested for the inhibition of hTOP3B relaxation activity. Five compounds with comparable IC_50_ to that of bemcentinib for the inhibition of hTOP3B were identified. These results suggest that the exploration of tyrosine kinase inhibitors and their analogs may allow the identification of novel potential topoisomerase inhibitors.

## 1. Introduction

Topoisomerases are essential enzymes required for controlling DNA supercoiling and untangling DNA during vital cellular processes including replication, transcription, and repair [1,2]. The change in DNA topology is brought about by the cutting and rejoining of DNA via a covalent protein DNA complex formed between the cleaved DNA and an active site tyrosine [3,4]. Type I topoisomerases cut and rejoin a single strand of DNA during catalysis while type II topoisomerase cut and rejoin a double-strand of DNA. These two classes of topoisomerases are further divided into subfamilies based on the similarity of the protein sequence and catalytic mechanism. In humans, there are two type IIA topoisomerases (TOP2A, TOP2B), one type IB topoisomerase (TOP1), and two type IA topoisomerases (TOP3A, TOP3B). The type IA and type IB topoisomerases are distinctly different in structure and mechanism. Type IA and type IIA topoisomerases form a 5′-phosphoryl tyrosine covalent intermediate and require a divalent ion for catalysis, while type IB topoisomerases form a 3′-phosphoryl tyrosine covalent intermediate and do not require a divalent ion for catalysis [4,5]. There is no sequence or structural homology between type IA and type IB topoisomerases [6,7]. A small molecule inhibitor with good selectivity for type IA topoisomerases should not inhibit type IB topoisomerases with similar potency.

Topoisomerase III beta (TOP3B) is the only RNA topoisomerase found in humans that can change the topology of both DNA and RNA substrates [8,9] for the regulation of genome stability, gene transcription, as well as mRNA translation and turnover [10,11,12,13]. TOP3B has been shown to be required for the efficient replication of many positive-sense single-stranded RNA viruses including flaviviruses (DENV, ZIKV and YFV) [14]. The Dengue virus (DENV) infects up to 400 million people each year as a serious global epidemic with no specific treatment available [15]. The host TOP3B could potentially be hijacked by RNA viruses to assist in viral protein translation or viral RNA transport and packaging in different stages of the viral life cycle [16,17]. In addition to use for antiviral therapy, inhibitors of human TOP3B could potentially be useful for anticancer treatment [18]. Many chemotherapeutic agents used in clinical settings are topoisomerase poisons that trap the covalent intermediate formed between topoisomerases and chromosomal DNA to induce cancer cell death [19,20,21]. Specific small molecule inhibitors of type IA topoisomerases that can be used in clinical settings remain to be identified. A previous high-throughput screening has identified a bisacridine compound and a thiacyanine compound that can act as TOP3B poison by acting on RNA to increase the level of the TOP3B covalent complex [22]. However, the inhibition of the complete cycle of TOP3B catalytic activity by these TOP3B poisons has not been demonstrated for these bisacridine and thiacyanine compounds. In this study, we first explored drug repurposing by attempting to identify inhibitors that can target human TOP3B through the virtual screening of a library of approved drugs or drug candidates in clinical development. We confirmed experimentally that the tyrosine kinase inhibitor bemcentinib [23,24] can inhibit the catalytic activity of human TOP3B. We then tested additional analogs of bemcentinib for the inhibition of TOP3B to compare the potency and selectivity.

## 2. Results

### 2.1. Hits from Virtual Screening of Library of Compounds That Are FDA-Approved or in Clinical Trials

Molecular docking was performed for 3855 compounds that are either FDA-approved or in clinical trials against 100 conformations of the TOP3B-RNA covalent complex generated by molecular dynamics simulation. The compounds with the top affinity scores are shown in Table 1. The list reveals that tyrosine kinase inhibitors, with the suffix -tinib, score highly in the computational screening. This is possibly due to the fact that topoisomerases impart their phosphoryl transfer activity through a catalytic tyrosine. These compounds are mostly inhibitors of growth factor receptors (GFRs), such as VEGFR-2, and are currently in trial for various cancer treatments [25].

### 2.2. Inhibition of Human TOP3B Relaxation Activity by Bemcentinib

**Figure 1:**
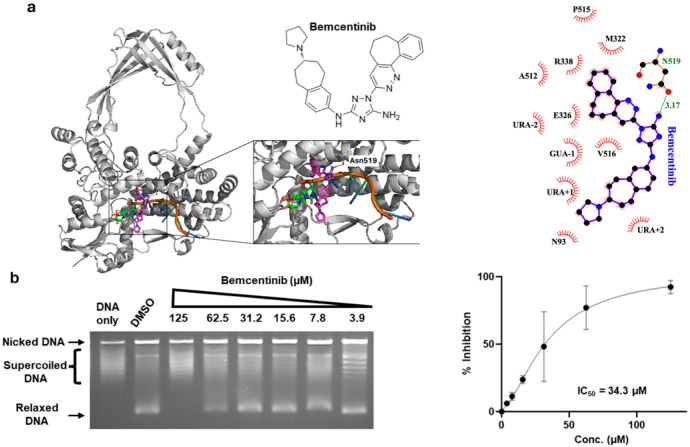
Identification of bemcentinib as a TOP3B inhibitor. (**a**) The structure of bemcentinib and its docking pose in the target site of TOP3B. Right: Interaction map generated with LigPlot+ for the bemcentinib in its binding site; (**b**) Chloroquine gel electrophoresis assay of TOP3B relaxation activity in the presence of the DMSO negative control or serial dilutions of bemcentinib. No enzyme is added to the DNA-only lane. The mean and standard deviation of the % of inhibition from four repeated experiments were plotted with GraphPad Prism version 8.4.2 to obtain the IC_50_ value for bemcentinib. The mean and standard deviation of the % of inhibition from four repeated experiments were plotted with GraphPad Prism version 8.4.2 to obtain the IC_50_ value for bemcentinib.

From the ranked compounds, we purchased the tyrosine kinase inhibitors golvatinib, bemcentinib, tucatinib, nilotinib, rebastinib, and radotinib to test at up to 200 µM concentrations for inhibition of the DNA relaxation activity of human TOP3B. Chloroquine was included in the gel electrophoresis buffer to separate the partially relaxed DNA product from the input supercoiled DNA. Inhibition was observed only for bemcentinib (Figure 1 and Figure S1). Figure 1a displays the best pose of bemcentinib docked near the binding pocket of TOP3B. To identify the interactions between bemcentinib and TOP3B, we generated a 2-D interaction map, which shows a polar interaction (3.17 Å) between the amide of the 1,2,4-triazole ring of the bemcentinib with the sidechain of Asn519 (Figure 1a, right). We also observed a hydrophobic interaction with other nearby residues and the nucleotides towards the 3′ end of the RNA. From serial dilutions, the IC_50_ of bemcentinib was measured to be 34.3 µM (Figure 1b).

### 2.3. Testing of Additional Small Molecules for Inhibition of Human TOP3B Relaxation Activity

**Figure 2:**
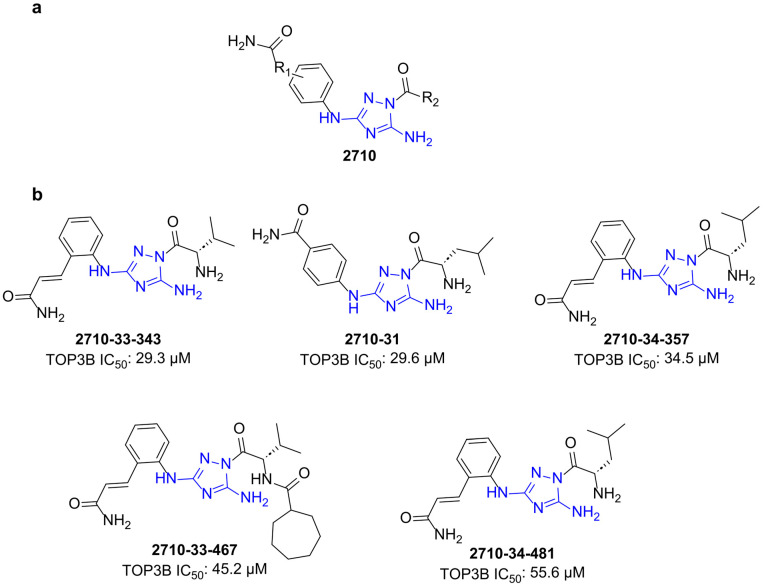
Structure of 2710 series of compounds. (**a**) Scaffold structure with the *N*^5^,*N*^3^-1*H*-1,2,4-triazole-3,5-diamine moiety. (**b**) The 2710 derivatives found to inhibit TOP3B with IC_50_ < 60 µM.

**Figure 3:**
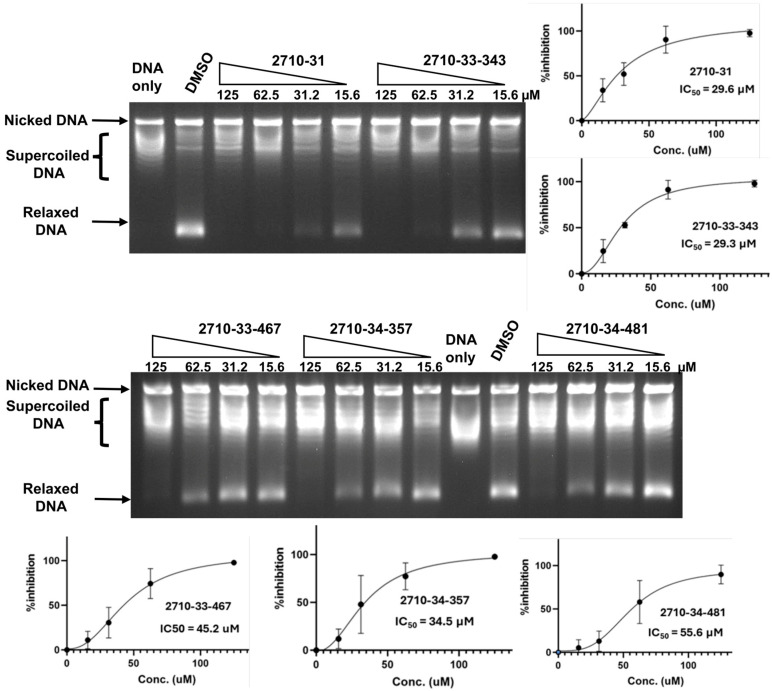
Inhibition of TOP3B relaxation activity by selected compounds from the 2710 series. Serial dilution of compounds from 125 to 15.6 µM were assayed by gel electrophoresis in the presence of chloroquine. No enzyme is added to the DNA-only lane. DMSO is used as the negative control for enzyme inhibition. The mean and standard deviation of the % of inhibition from three repeated experiments were plotted with GraphPad Prism version 8.4.2 to obtain the IC_50_ values.

We synthesized a set of compounds under the 2710 series that have the *N*^5^,*N*^3^-1*H*-1,2,4-triazole-3,5-diamine moiety of bemcentinib (Figure 2a). These compounds (Figure S2) were first tested at 200 µM concentrations for the inhibition of the human TOP3B relaxation activity. Compounds that showed complete inhibition at 200 µM (Figure S3) were tested further at lower concentrations. We found that the compounds shown in Figure 2b could inhibit TOP3B with an IC_50_ < 60 µM (Figure 3).

### 2.4. Comparison of Selectivity for Inhibition of Human TOP3B Versus TOP1

**Figure 4:**
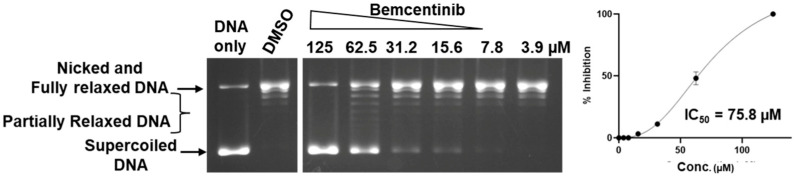
Inhibition of human TOP1 relaxation activity by bemcentinib. The lanes shown are from the same gel. No enzyme is added to the DNA-only lane. DMSO is used as the negative control for enzyme inhibition. The mean and standard deviation from three repeated experiments were plotted with GraphPad Prism version 8.4.2 to obtain the IC_50_ value.

**Figure 5:**
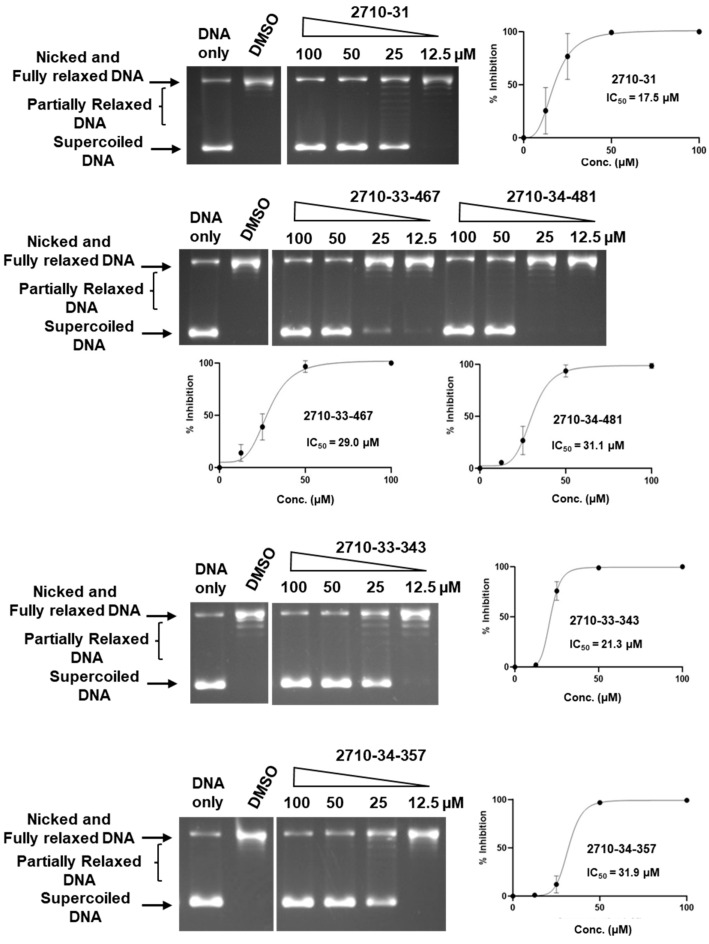
Inhibition of human TOP1 relaxation activity by selected 2710 derivatives. The lanes shown for each compound are from the same gel. No enzyme is added to the DNA-only lane. DMSO is used as the negative control for enzyme inhibition. The mean and standard deviation of the % of inhibition from three repeated experiments were plotted with GraphPad Prism version 8.4.2 to obtain the IC_50_ value.

To evaluate the selectivity of bemcentinib and the identified 2710-series compounds as topoisomerase inhibitors, they were also tested for the inhibition of type IB human TOP1 which has a distinctly different structure and mechanism from the type IA topoisomerase TOP3B. Chloroquine is not required to observe the relaxation of supercoiled DNA by TOP1 by gel electrophoresis because the relaxation of supercoiled DNA by TOP1 continues after initial removal of negative supercoils while TOP3B relaxation would not proceed further when the DNA substrate no longer possesses a significant single-stranded region. The results of the TOP1 assay (Figure 4 and Figure 5) showed that only bemcentinib inhibits TOP3B with greater potency than TOP1 (Table 2). The IC_50_ values for TOP1 inhibition by the 2710 compounds were all lower than the IC_50_ values for TOP3B inhibition.

**Figure 6:**
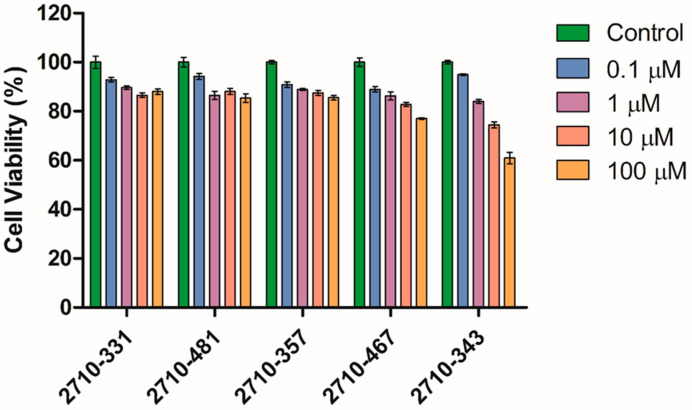
Cell viability (%) of HdFa in response to log concentrations of inhibitors from the 2710 series (n = 4, mean ± SD).

The cytotoxic potential of the newly synthesized topoisomerase inhibitors was evaluated using primary human dermal fibroblasts (HdFa) across a range of log concentrations, up to 100 µM (Figure 6). All tested compounds demonstrated minimal cytotoxicity, maintaining cell viabilities between 75% and 85% at the highest concentration tested (100 µM), with the exception of compound 2710-343, which exhibited a comparatively lower cell viability of ~60% at 100 µM. These results indicate a generally favorable safety profile for most of these compounds in non-cancerous fibroblasts. However, compound 2710-343 may warrant closer evaluation for potential off-target effects or structural cytotoxic liabilities.

## 3. Discussion

TOP3B is the only human topoisomerase that can change the topology of RNA. Based on a previous report on the requirement of human TOP3B for the efficient replication of positive-sense ssRNA viruses [14], we conducted virtual screening to try to identify inhibitors of TOP3B from 3855 approved drugs and drug candidates as potential antiviral candidates. Notably, most of the top virtual screening hits are inhibitors of tyrosine protein kinases. Topoisomerases share similarity with tyrosine protein kinases in likely interactions with a nucleotide moiety as part of ATP for the kinases and part of nucleic acid substrates for topoisomerases, as well as catalysis of Mg(II)-dependent phosphoryl transfer to tyrosine in the reaction mechanism. Dual inhibition of type IA/IIA topoisomerases and tyrosine kinases by small molecules has been reported previously [26,27]. In vitro biochemical assay of six purchasable tyrosine kinase inhibitors identified as TOP3B inhibitor candidates in the virtual screening showed that bemcentinib which targets the AXL receptor tyrosine kinase can inhibit the relaxation activity of TOP3B with an IC_50_ of 34.3 µM. The other five virtual screening hits tested experimentally did not show inhibition in the assay at 200 µM. A more advanced virtual screening methodology may increase the success rate of future virtual screening projects for TOP3B inhibitors. While it is logical to target the nucleic acid binding sites of topoisomerases for virtual inhibitor screening, these binding sites and interactions involved may not favor specific inhibition by small molecules. Alternative allosteric sites or hinges for conformational change in the topoisomerase could be explored in future virtual screening attempts. TDRD3 has been shown to bind to the hinge region between domains D2 and D4 for a potential role in supporting the gate opening and closing dynamics [28,29]. Therefore it should also be noted that since the TDRD3 protein is present in our TOP3B assay for enhancing the TOP3B relaxation activity, we cannot rule out that the inhibitors may interfere with the TDRD3 enhancement to affect the TOP3B relaxation activity observed here.

We further explored TOP3B inhibition by 2710 derivatives that possess the *N*^5^,*N*^3^-1*H*-1,2,4-triazole-3,5-diamine moiety present in bemcentinib. Many of the 2710 derivatives tested showed a detectable inhibition of TOP3B relaxation activity at 200 µM, and five of the compounds can inhibit TOP3B with an IC_50_ < 60 µM. All identified active compounds possess hydrophobic aliphatic side chains such as isopropyl and isobutyl groups at the R2 position. However, these five compounds were found to also inhibit TOP1 relaxation activity with a lower IC_50_. While bemcentinib and the 2710 derived inhibitors have the same 1*H*-1,2,4-triazole-3,5-diamine scaffold, the nature of substituents at N3 and N1are different. It seems that bulky substituted aryl compounds favor inhibitory activity against TOP3B compared to TOP1. The potency and selectivity for TOP3B inhibition could potentially be further improved by exploring other analogs of bemcentinib. Exploring additional scaffolds of tyrosine kinase inhibitors may also lead to the identification of novel TOP3B or TOP1 inhibitors. More selective TOP3B inhibitors would be useful as tools for basic studies of TOP3B cellular functions or leads for the development of antiviral therapy against flaviviruses that have no currently available treatment options. A previous study using TOP3B knockout cells showed that TOP3B is required for the efficient replication of dengue and chikungunya viruses [14]. For TOP3B inhibitors to achieve an antiviral effect, the TOP3B inhibitors would probably need to inhibit the catalytic activity of TOP3B. While the bisacridine and thiacyanine compounds characterized in an earlier study act as topoisomerase poisons against TOP3B with a mechanism that may be favored for anticancer application, these two TOP3B poisons have not been shown to inhibit the topological change resulting from the TOP3B catalytic activity [22]. The potential of the 2710 derivatives identified to be human TOP1 inhibitors could also be further explored for their use as TOP1 poisons for anticancer therapy [19,30] or TOP1 catalytic inhibitors for preventing death from lethal inflammation [31,32].

## 4. Materials and Methods

### 4.1. Molecular Dynamics Simulation

The TOP3B-RNA covalent complex was generated by first obtaining the apo-structure of the human TOP3B from the protein data bank (PDB ID: 5GVC) [28]. A single-stranded RNA with the sequence 5′-AAACUG↓UU-3′ (based on the sequence of a previously reported TOP3B RNA cleavage oligonucleotide substrate [9]) was placed near the active site of the crystal structure. Next, a covalent bond between the catalytic tyrosine Y336 and the 5′-phosphate of the U nucleotide downstream of the cleavage site in the RNA was computationally generated using a method described previously [33], thus creating a model of the covalently bonded structure of the TOP3B enzyme (hTOP3Bcc). Conformations were then generated with all-atom molecular dynamics (MD) simulation using a standard MD procedure that included possible protein conformational flexibility. The system was prepared using the CHARMM-GUI web interface [34]. The hTOP3Bcc structure (with a net charge of −10) was solvated in a cubic box of dimension 127 × 127 × 127 Å3 with TIP3 water and neutralized with a NaCl concentration of 0.15 M by adding 183 Na^+^ and 173 Cl^−^ ions. The resulting system comprised 192,807 atoms. MD simulation was performed with NAMD 2.13 [35] using the CHARMM36m [36] force field. The particle mesh Ewald (PME) [37] method was used for long-range electrostatic interactions with periodic boundary conditions and a non-bonded cut-off set at 12 Å, while the SHAKE [38] algorithm was used for constraining hydrogen atom covalent bonds. The Nose–Hoover Langevin-piston barostat method with a piston period of 50 fs and a decay of 25 fs was used for pressure control [39] and the Langevin temperature-coupling thermostat with a friction coefficient of 1 ps^−1^ was used for temperature control. The prepared system was minimized for 10,000 steps using CHARMM36m force field parameter files and a TYDN.prm file. Next, the system was equilibrated at 303.15 K for 100 ps with a 1 fs time step with protein heavy atoms harmonically restrained. Finally, a 500 ns NPT (constant pressure, temperature) production run was performed at 303.15 K.

### 4.2. Molecular Docking of Compounds

To prepare the receptor structures for virtual screening, 100 protein conformations were extracted from the production run using VMD [40]. Extracted pdb files were then converted to pdbqt format using AutoDockTools 4.2 [41].

The 3D structures of 3855 FDA-approved drugs and drugs undergoing clinical trials were obtained in the SDF format from the DrugBank 5.0 database [42]. Then, Open Babel [43] was used to convert the SDF to pdbqt format with a three dimensional (3D) structure and added polar hydrogen atoms. A cavity that overlaps with the expected nucleic acid binding region near the active site of TOP3B was selected as the target site for the virtual screening, with a box size of 21Å × 25Å × 24Å. Vina from AutoDockTools 4.2 [41] was used to perform docking and screening. Top hits were sorted and ranked based on their binding energy scores using custom scripts. PyMol (The PyMOL Molecular Graphics System, Version 3.0 Schrödinger, LLC) was used for the visualization of the best docked pose of bemcentinib. LigPlot^+^ (v2.2) [44] was used to generate the interaction map between bemcentinib and TOP3B using the best docked pose.

### 4.3. Assay of TOP3B Activity Inhibition

Golvatinib, bemcentinib, tucatinib, nilotinib, rebastinib, and radotinib were purchased from Adooq BioScience. The identity and purity of compounds (>95%) were authenticated by NMR and LC-MS.

Human TOP3B protein was obtained through custom production by Genscript. The coding sequence of human TOP3B was custom synthesized by Genscript and cloned into vector pFastBacGST to generate the recombinant baculovirus expressing human TOP3B protein with the N-terminal GST tag and C-terminal 6xHis tag. Following expression in sf9 insect cells, the recombinant TOP3B protein was purified to >95% homogeneity by Genscript using GST column and superdex 200 16/600G chromatography. A buffer containing 20 mM of Tris-HCl (pH 7.5), 750 mM of KCl, 10% glycerol, 1 mM of EDTA, 0.05% NP-40, and 1mM of DTT was used for storage and dilution.

The presence of the TDRD3 protein enhances the relaxation activity of human TOP3B through the direct binding of the proposed TOP3B hinge region for gate opening and closing [28,29]. The human *TDRD3* coding sequence was amplified by PCR from the TDRD3 clone in pEGFP-C1 [45] using a forward primer of 5′-ATGGCCCAGGTGGCCGGC-3′ and a reverse primer of 5′-TTAGTTCCGAGCCCGGGGTGG-3′. The amplified *TDRD3* gene was joined with the backbone of the DHFR-His plasmid (from New England BioLab) using the NEB HiFi assembly kit with the forward primer 5′-GGAGGATCCCGGGAATTC-3′ and the reverse primer 5′-GGATCCGTGGTGATGGTG-3′ so that the *TDRD3* gene replaced the *DHFR* gene. The his-tagged TDRD3 protein was expressed from the T7 promoter in *E. coli* Rosetta(DE3) cells and purified using the HisPur Ni-NTA spin column from Thermo Fisher according to the manufacturer’s protocol.

Inhibition of the relaxation activity of human TOP3B was carried out in a reaction buffer containing 20 mM of HEPES-KOH, with a pH of 7.5, 1 mM of DTT, 0.1 mg/mL of BSA, and 5 mM of MgCl_2_. TOP3B and TDRD3 were added at 18 nM to each reaction mix followed by 0.5 μL of DMSO or indicated concentrations of compounds, and lastly by the addition of 500 ng of supercoiled pBAD/Thio plasmid DNA (purified by CsCl gradient centrifugation) for a final reaction volume of 20 μL. After mixing by gentle vortex, the reaction mixtures were spun down and incubated at 37 °C for 1 h. The relaxation reactions were terminated by the addition of 1 μL of 10% SDS and 2 μL of 800 units/mL of proteinase K (New England Biolabs) and incubated at 45 °C for 30 min. Following the addition of 5 μL of stop solution (50 mM of EDTA, 50% glycerol, and 0.5% *v/v* bromophenol blue), the reaction products were analyzed in a 1% agarose gel with TAE (40 mM of Tris-acetate, pH 8.0, 2 mM of EDTA) buffer containing 5 μg/mL of chloroquine at 25 V (1 V/cm) for 18 h. Gels were washed with TAE buffer for 1 h and 50 mM of NaCl for 1 h to remove the chloroquine, and finally with deionized water for 30 min. Gels were stained for 2 h with 1 μg/mL of SYBR Gold solution (Thermo Fischer Scientific, Waltham, MA, USA). Gels were destained with deionized water for 15 min and photographed over UV light with the AlphaImager system. The amount of the relaxed DNA produced by TOP3B in each reaction was quantified using the AlphaImager software version 1.5.0 to calculate the percentage of inhibition versus the DMSO control.

### 4.4. Assay of Inhibition of Human TOP1 Relaxation Activity

Recombinant human TOP1 was expressed from clone pYES2-TOP1 transformed into *Saccharomyces cerevisiae strain* EKY3 [46]. Inhibition of TOP1 relaxation activity was assayed as described [46] in a 20 µL reaction containing 200 ng of supercoiled pBAD/Thio plasmid DNA and 1 unit of enzyme in 10 mM of Tris-HCl, with a pH of 8.0, 1 mM of EDTA, 150 mM of NaCl, 0.1 mM of spermidine, 0.1 mg/mL of BSA, and 5% glycerol. The reactions were incubated at 37 °C for 30 min before the addition of 4 µL of stop solution (6% SDS, 0.6% (*wv*/*v*)) bromophenol blue and 40% glycerol. Gel electrophoresis for the separation of the supercoiled DNA substrate from the relaxed DNA was conducted in 1% agarose gel with TAE buffer for 20 h at 25 V. The DNA was stained with ethidium bromide for an hour and then soaked in water for 5 min. Gels were photographed over UV light with the AlphaImager system. The amount of the supercoiled DNA substrate remaining in each reaction was quantified using the AlphaImager software to calculate the percentage of inhibition versus the DMSO control.

### 4.5. Synthesis of Small Molecules with Disubstituted N^5^,N^3^-1H-1,2,4-Triazole-3,5-Diamine

**Scheme 1:**
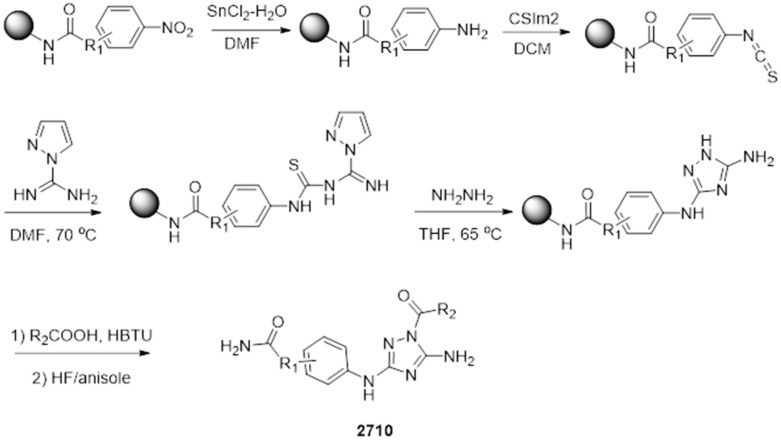
Synthetic strategy of disubstituted *N*^5^,*N*^3^-1*H*-1,2,4-triazole-3,5-diamine derivatives **2710**.

The parallel synthesis of all compounds was performed using the previously described “tea-bag” technology [47,48]. The synthetic strategy for the 2710 series of compounds is shown in Scheme 1. Starting from preprepared MBHA resin bound nitro aryl compounds, the nitro group was reduced in the presence of tin chloride (SnCl_2_·2H_2_O) and then washed with DMF (10 times) and the resulting aryl amines were treated with thiocarbonyldiimidazole (CSIm_2_) in anhydrous DCM. Following decantation, the generated aryl isothiocyanate was treated with 1*H*-Pyrazole-1-carboxamidine hydrochloride overnight in anhydrous DMF at 70 °C. The resulting thiourea intermediate was treated with hydrazine (3 eq) overnight in anhydrous THF at 65 °C to generate the corresponding substituted *N*^3^-phenyl-1*H*-1,2,4-triazole-3,5-diamine. Using well-described procedures, the acylation of the obtained compounds with different carboxylic acids in the presence of HBTU (4 eq) and DIEA (4eq) for 4 h led to the desired disubstituted *N*^5^,*N*^3^-1*H*-1,2,4-triazole-3,5-diamine derivatives [49]. The final compounds were cleaved from the solid support in the presence of HF/anisole (95:5), extracted with acetic acid, and underwent three cycles of freezing and lyophilizing. The crude products were purified by RP-HPLC, and the desired compounds were obtained with a purity higher than 85% as determined by LC-MS shown in Figure S4 of the supporting information, along with HRMS data.

### 4.6. Dose Response of 2710-Inhibitors on Cell Viability of Human Dermal Fibroblasts (HDFa)

Human dermal fibroblasts (HdFa; PCS-210-012™, ATCC, Manassas, VA, USA) were cultured in Dulbecco’s Modified Eagle Medium (DMEM; supplemented with GlutaMAX™ and sodium pyruvate; Gibco, Waltham, MA, USA), containing 10% fetal bovine serum (FBS; Gibco) and 1% penicillin-streptomycin (10,000 U/mL; Gibco) [50,51]. Cells were maintained in T75 tissue culture flasks (Thermo Scientific Nunc™, Roskilde, Denmark) under standard incubation conditions (37 °C, 5% CO_2_, humidified atmosphere). Upon reaching confluency, cells were harvested and seeded into black-walled, clear-bottom, 96-well plates at a density of 5000 cells/well in 100 µL of complete growth medium. Following overnight incubation, the cells reached approximately 80–90% confluence.

The culture medium was then replaced with 100 µL of test compounds (2710-331, 2710-481, 2710-357, 2710-476, and 2710-343) prepared at concentrations of 0.1 µM, 1 µM, 10 µM, and 100 µM (n = 4 wells per condition). All compounds were initially dissolved in DMSO and subsequently diluted in serum-free DMEM to achieve a final DMSO concentration ≤ 0.1% (*v*/*v*). Untreated cells (receiving only medium) served as negative controls.

After 24 h of compound exposure, 20 µL of CellTiter-Blue^®^ reagent (Promega, WI, USA) was added to each well. Plates were incubated for an additional 2 h at 37 °C, after which the fluorescence of the metabolized resorufin product was measured using a Synergy Multi-Mode Plate Reader (BioTek Instruments, Winooski, VT, USA) at an excitation wavelength of 560 nm and an emission wavelength of 590 nm. Cellular viability in response to the test compounds was expressed as a percentage relative to the untreated control group.

(1) $$Cell\;viability\;(\%)=\frac{\mathrm{Fluorescence}\mathrm{intensity}\mathrm{of}\mathrm{treated}\mathrm{cells}}{\mathrm{Fluorescence}\mathrm{intensity}\mathrm{of}\mathrm{control}\mathrm{cells}}\times 10$$

## 5. Conclusions

In this study, we first identified the tyrosine kinase inhibitor bemcentinib as a TOP3B inhibitor from the virtual screening of a library of approved drugs and drug candidates. This is the first time that a small molecule has been shown to inhibit the topological change resulting from the catalytic activity of TOP3B. After synthesizing and testing the analogs with a similar scaffold, we identified additional TOP3B inhibitors that also inhibit human TOP1 with less selectivity than bemcentinib. The results from this study suggest that investigation of tyrosine kinase inhibitor candidates may yield novel topoisomerase inhibitors. Therapeutic applications for the TOP3B inhibitors would require improving their potency and demonstrating their selectivity against both type IB and type IIA human topoisomerases. Further development and improvement of catalytic activity assays available is needed for such future studies to allow for kinetic measurements and the less-variable determination of catalytic activity. The performance of our DNA relaxation assay is limited by the relatively low yield of the relaxed DNA product formed from TOP3B catalysis so reaction parameters should be further optimized if possible. The initial in silico screening using a limited number of conformations limits the identification of an ideal inhibitor-bound complex. A more exhaustive computational sampling of the receptor conformations and recent AI-based tools may better predict the binding of inhibitor candidates. Also, the use of other PDB structures with different substrate-bound TOP3B conformations (e.g., 9C9Y and 9CAG) may broaden the sampling of the receptor conformations. Ultimately, a structure of a TOP3B–ligand complex determined by X-ray crystallography or cryo-EM would validate the computational results and greatly aid the design of improved hits. The characterization of TOP3B inhibitors would also be much more relevant physiologically if cellular studies and the catalytic activity assay for change in RNA topology are included in future studies. RNA substrates that are suitable for the measurement of TOP3B RNA topoisomerase catalytic activity would need to be more readily available at reasonable costs for extensive use in inhibitor assays.

## Figures and Tables

**Table 1:** List of compounds obtained after virtual screening of drugs that are FDA-approved or undergoing clinical trial against TOP3B.

Affinity Score (kcal/mol)	Compound	Known Activities (Drug Bank Description)
−14.0	Golvatinib	Dual kinase inhibitor of c-Met (hepatocyte growth factor receptor) and VEGFR-2 (vascular endothelial growth factor receptor).
−13.8	Bemcentinib	Orally available and selective inhibitor of the AXL receptor tyrosine kinase (UFO), with potential antineoplastic activity.
−13.6	Anatibant	Selective and potent Bradykinin B2 receptor antagonist developed for the treatment of traumatic brain injury (TBI).
−13.4	Tucatinib	Tyrosine kinase inhibitor that targets the epidermal growth factor receptor 2 (HER2), used in combination therapy against refractory, advanced or metastatic HER2-positive breast and colorectal cancer.
−13.4	Phthalocyanine	A dye currently under investigation for treating patients with actinic keratosis, Bowen’s disease, skin cancer, or stage I or stage II mycosis fungoides.
−13.3	Conivaptan	Arginine vasopressin (AVP) receptor antagonist with affinity for AVP receptor subtypes V1A and V2, approved for hyponatremia (low blood sodium levels) caused by the syndrome of inappropriate antidiuretic hormone (SIADH).
−13.1	Nilotinib	Tyrosine kinase inhibitor under investigation as a possible treatment for chronic myelogenous leukemia (CML).
−13.0	Rebastinib	Orally bioavailable inhibitor of multiple tyrosine kinases with potential antineoplastic activity.
−12.9	Radotinib	Second-generation tyrosine kinase inhibitor of Bcr-Abl fusion protein and the platelet-derived growth factor receptor (PDGFR), with potential antineoplastic activity.

**Table 2:** IC_50_ values and 95% confidence intervals for the inhibition of human TOP3B and TOP1 relaxation activity by bemcentinib and inhibitors from the 2710 series.

Compound	TOP3B IC_50_ µM	95% CI µM	TOP1 IC_50_ µM	95% CI µM
Bemcentinib	30.2	21.6–65.1	75.8	68.9–88.9
2710-31	29.6	7.8–41.2	17.5	13.6–23.1
2710-33-343	29.3	23.3–39.0	21.3	19.5–24.9
2710-33-467	45.2	32.8–59.8	29.0	25.0–33.2
2710-34-357	34.5	22.2–49.6	31.9	27.4–35.4
2710-34-481	55.6	40.1–74.2	31.1	22.89–34.62

## Data Availability

All data generated or analyzed during this study are included in this published article and its Supplementary Information files.

## Supplementary Materials

The following supporting information can be downloaded at https://www.mdpi.com/article/10.3390/ijms26136193/s1.
