# Supplementary material for: Substituted Triazole-3,5-Diamine Compounds as Novel Human Topoisomerase III Beta Inhibitors
Source: Int J Mol Sci. 2025 Jun 27;26(13):6193. doi: 10.3390/ijms26136193 (PMC12249905; doi:10.3390/ijms26136193)

Figure S1. Assay of inhibition of human TOP3B relaxation of negatively supercoiled DNA. Puchased hits from virtual screening were tested at concentrations of 200, 100 and 50 μM.

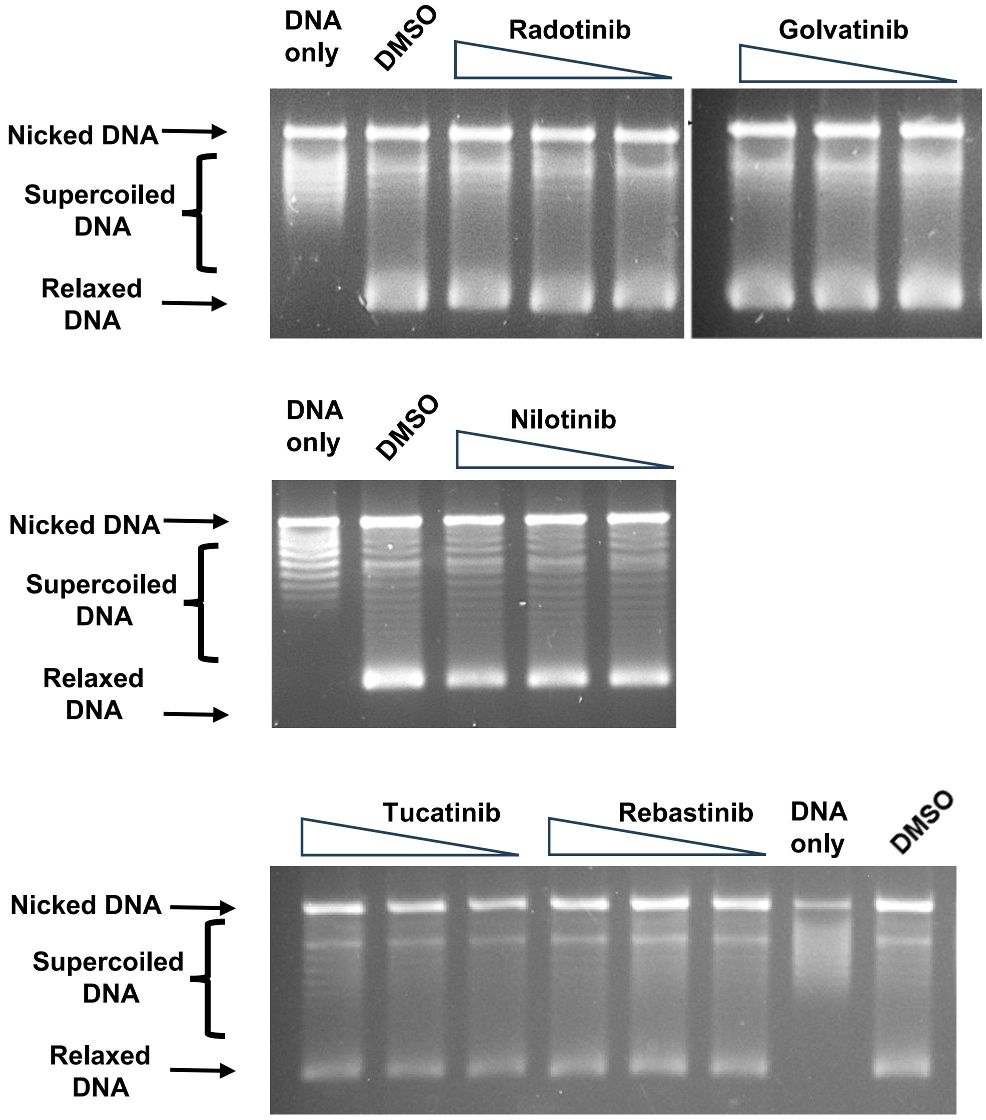

Figure S2. Structure, chemical formula and molecular weight of 2710 series of compounds tested in this study.

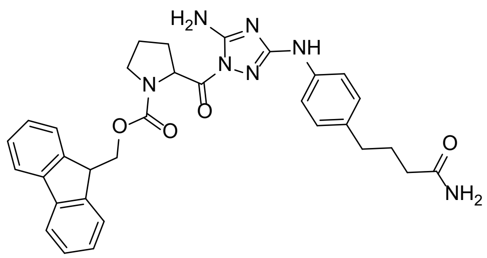

2710-1

Chemical Formula: $C_{32}H_{33}N_7O_4$
Molecular Weight: 579.66

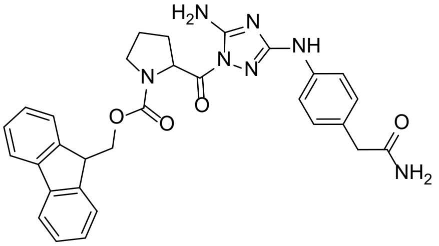

2710-2

Chemical Formula: $C_{30}H_{29}N_7O_4$
Molecular Weight: 551.61

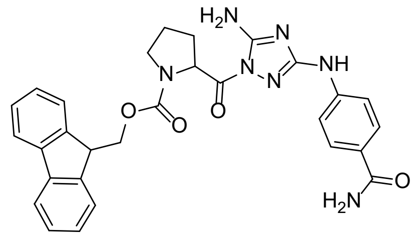

2710-3

Chemical Formula: $C_{29}H_{27}N_7O_4$
Molecular Weight: 537.58

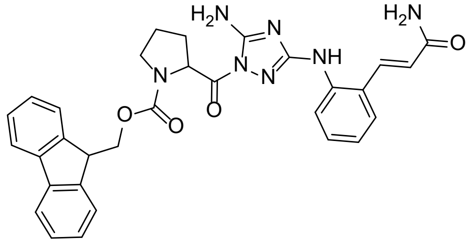

2710-4

Chemical Formula: $C_{31}H_{29}N_7O_4$
Molecular Weight: 563.62

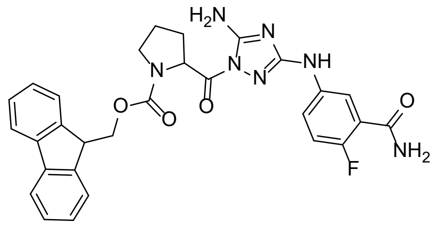

2710-6

Chemical Formula: $C_{29}H_{26}FN_7O_4$
Molecular Weight: 555.57

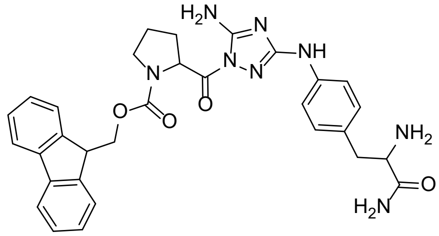

2710-9,10

Chemical Formula: $C_{31}H_{32}N_8O_4$
Molecular Weight: 580.65

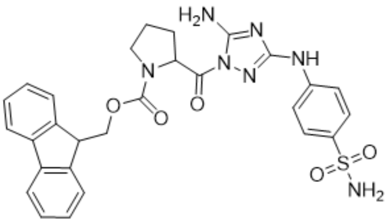

2710-11,12,13

Chemical Formula: $C_{28}H_{27}N_7O_5S$
Molecular Weight: 573.63

2710-14

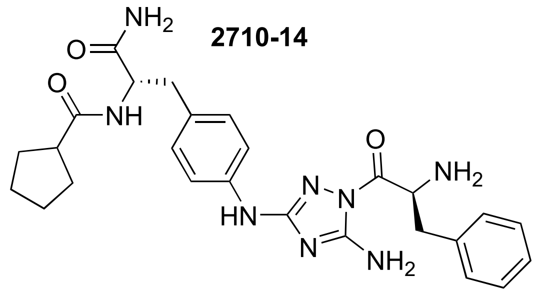

Chemical Formula: $C_{26}H_{32}N_8O_3$
Molecular Weight: 504.60

2710-15

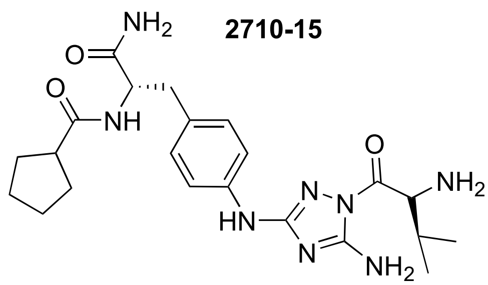

Chemical Formula: $C_{22}H_{32}N_8O_3$
Molecular Weight: 456.55

2710-16

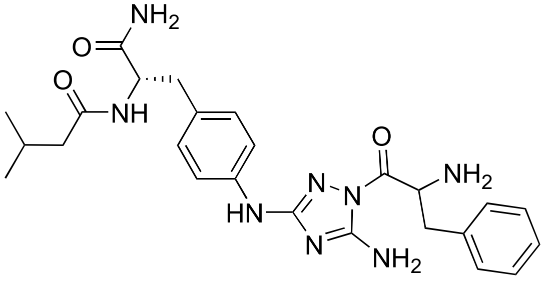

Chemical Formula: $C_{25}H_{32}N_8O_3$
Molecular Weight: 492.58

2710-17

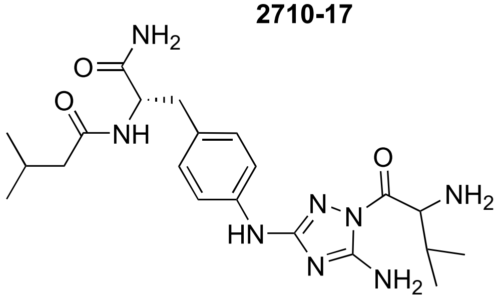

Chemical Formula: $C_{21}H_{32}N_8O_3$
Molecular Weight: 444.54

2710-18

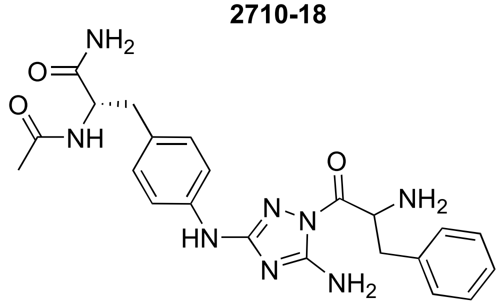

Chemical Formula: $C_{22}H_{26}N_8O_3$
Molecular Weight: 450.50

2710-19

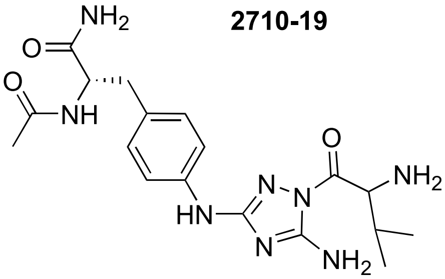

Chemical Formula: $C_{18}H_{26}N_8O_3$
Molecular Weight: 402.46

2710-20-401

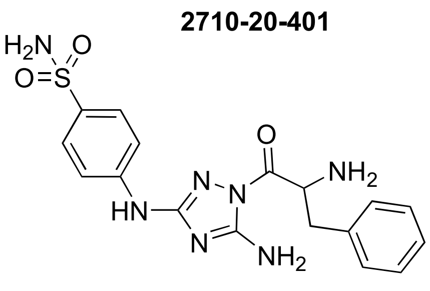

Chemical Formula: $C_{17}H_{19}N_7O_3S$
Molecular Weight: 401.45

2710-20-526

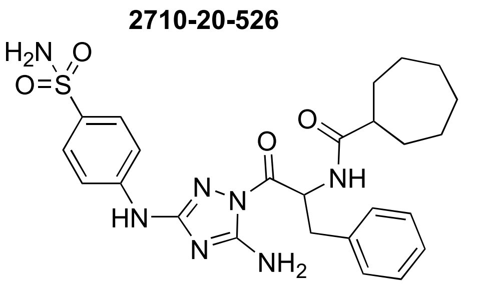

Chemical Formula: $C_{25}H_{31}N_7O_4S$
Molecular Weight: 525.63

2710-21

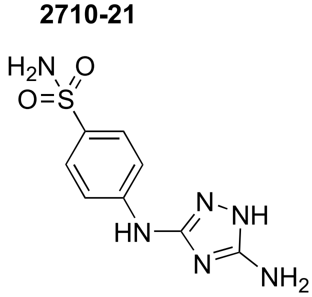

Chemical Formula: $C_8H_{10}N_6O_2S$
Molecular Weight: 254.27

2710-22

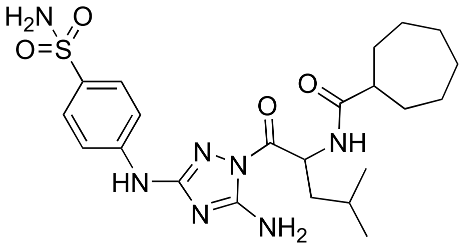

Chemical Formula: $C_{22}H_{33}N_7O_4S$
Molecular Weight: 491.61

2710-23

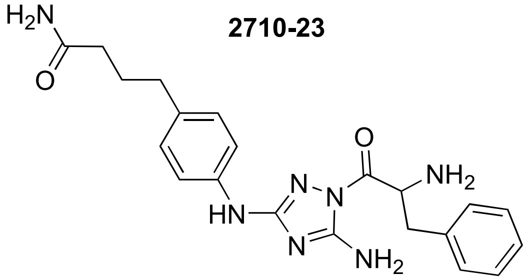

Chemical Formula: $C_{21}H_{25}N_7O_2$
Molecular Weight: 407.48

2710-24

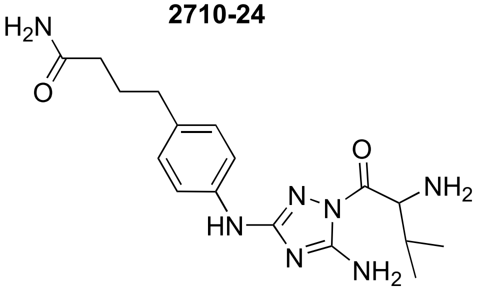

Chemical Formula: $C_{17}H_{25}N_7O_2$
Molecular Weight: 359.43

2710-25-498

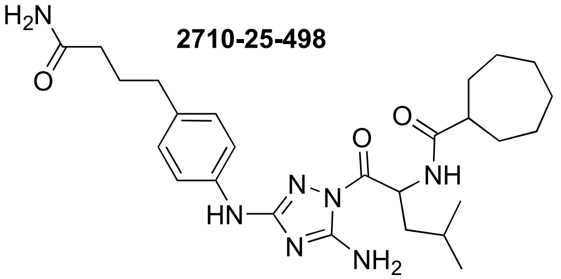

Chemical Formula: $C_{26}H_{39}N_7O_3$
Molecular Weight: 497.64

2710-25-374

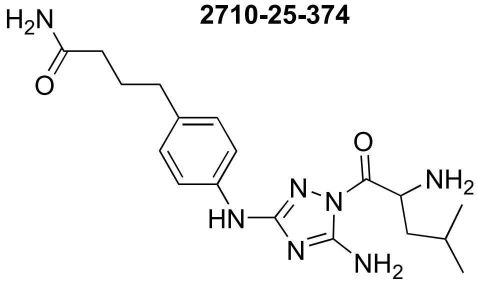

Chemical Formula: $C_{18}H_{27}N_7O_2$
Molecular Weight: 373.46

27110-26

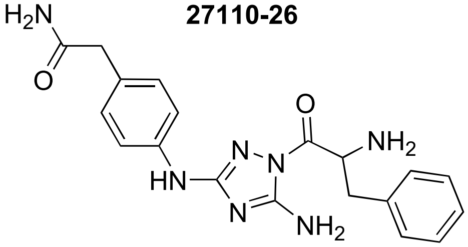

Chemical Formula: $C_{19}H_{21}N_7O_2$
Molecular Weight: 379.42

2710-27

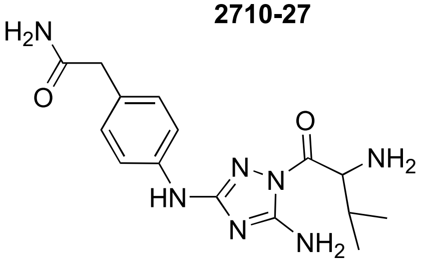

Chemical Formula: $C_{15}H_{21}N_7O_2$
Molecular Weight: 331.38

2710-28

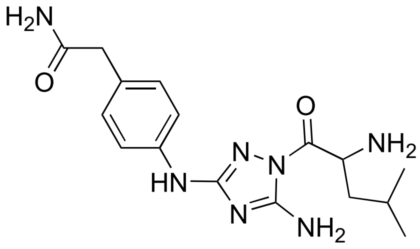

Chemical Formula: $C_{16}H_{23}N_7O_2$
Molecular Weight: 345.41

2710-30

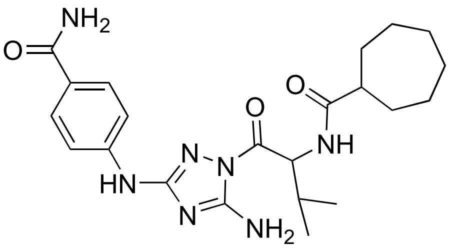

Chemical Formula: $C_{22}H_{31}N_{7}O_{3}$
Molecular Weight: 441.54

2710-31

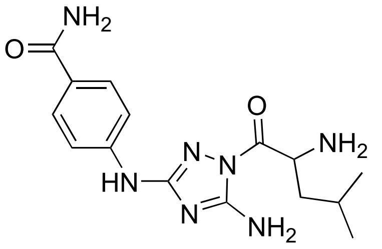

Chemical Formula: $C_{15}H_{21}N_{7}O_{2}$
Molecular Weight: 331.38

2710-32-391

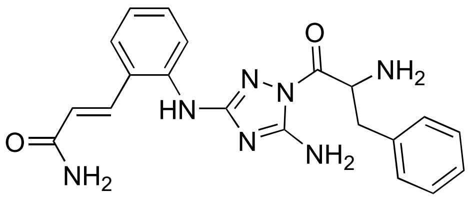

Chemical Formula: $C_{20}H_{21}N_{7}O_{2}$
Molecular Weight: 391.44

2710-32-516

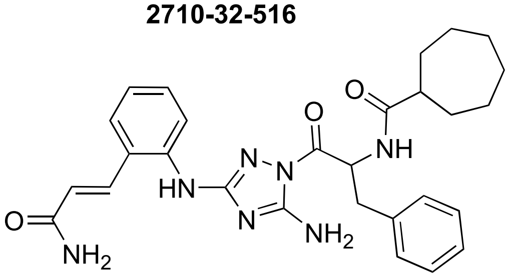

Chemical Formula: $C_{28}H_{33}N_{7}O_{3}$
Molecular Weight: 515.62

2710-33-343

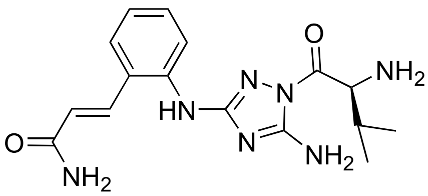

Chemical Formula: $C_{16}H_{21}N_{7}O_{2}$
Molecular Weight: 343.39

2710-33-468

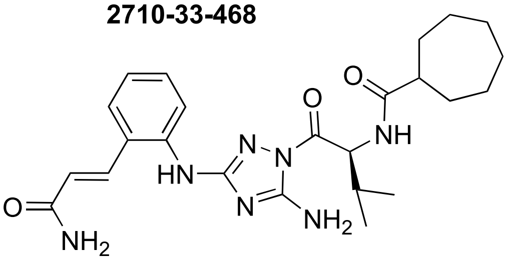

Chemical Formula: $C_{24}H_{33}N_{7}O_{3}$
Molecular Weight: 467.57

2710-34-357

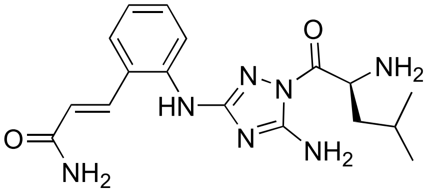

Chemical Formula: $C_{17}H_{23}N_{7}O_{2}$
Molecular Weight: 357.42

2710-34-481

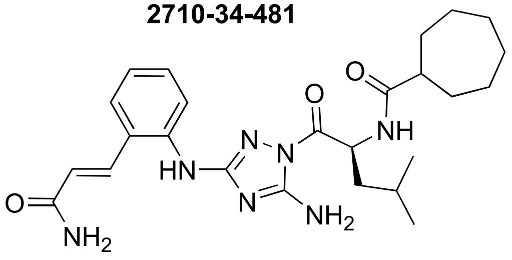

Chemical Formula: $C_{25}H_{35}N_{7}O_{3}$
Molecular Weight: 481.60

Figure S3. Assay of inhibition of human TOP3B relaxation of negatively supercoiled DNA by compounds in the 2710 series. The compounds were tested at concentration of (a) 200 μM (b) 125 and 62.5 μM. Each panel shows reactions analyzed on the same gel.

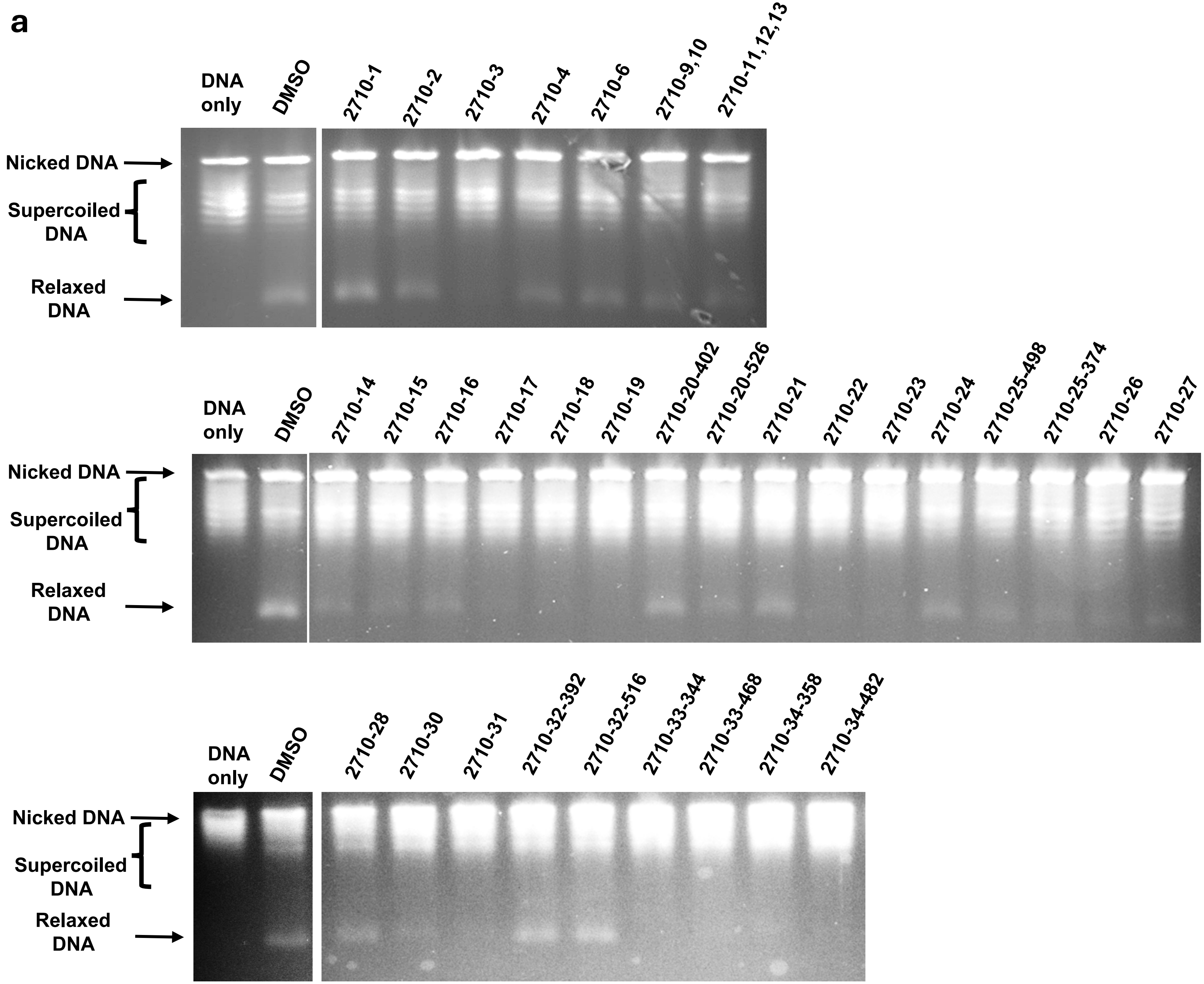

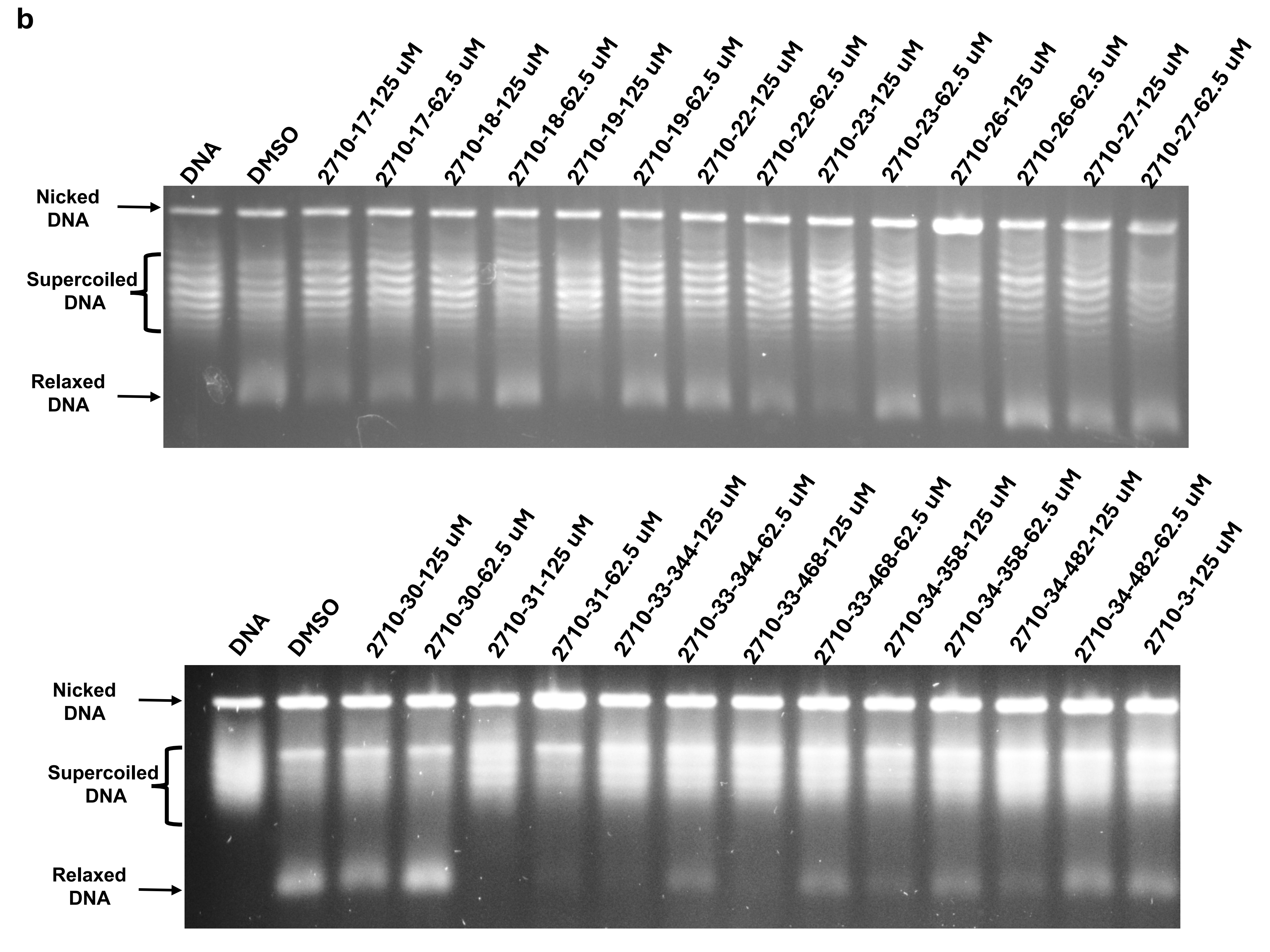
b
DNA
DMSO
2710-17-125 uM
2710-17-62.5 uM
2710-18-125 uM
2710-18-62.5 uM
2710-19-125 uM
2710-19-62.5 uM
2710-22-125 uM
2710-22-62.5 uM
2710-23-125 uM
2710-23-62.5 uM
2710-26-125 uM
2710-26-62.5 uM
2710-27-125 uM
2710-27-62.5 uM
Nicked DNA
Supercoiled DNA
Relaxed DNA
DNA
DMSO
2710-30-125 uM
2710-30-62.5 uM
2710-31-125 uM
2710-31-62.5 uM
2710-33-344-125 uM
2710-33-344-62.5 uM
2710-33-468-125 uM
2710-33-468-62.5 uM
2710-34-358-125 uM
2710-34-358-62.5 uM
2710-34-482-125 uM
2710-34-482-62.5 uM
2710-3-125 uM
Nicked DNA
Supercoiled DNA
Relaxed DNA

Figure S4. LC/MS of identified compounds 2710 series with strongest TOP3B inhibition and NMR data of representative compound 2710-19.

2710-31

(MW331)

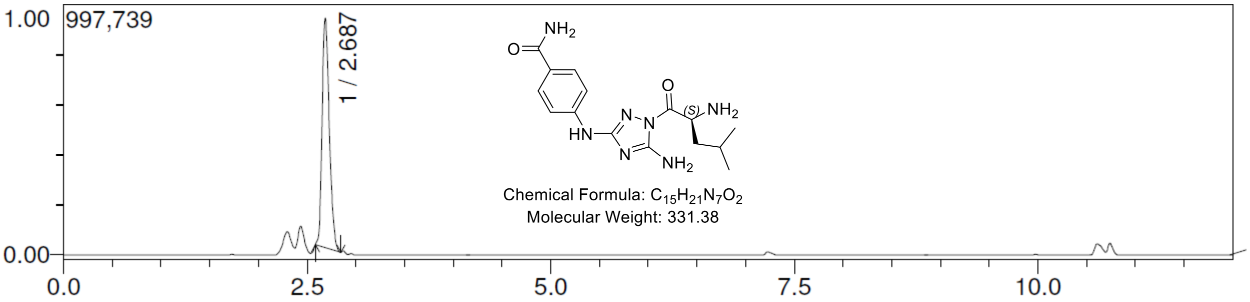

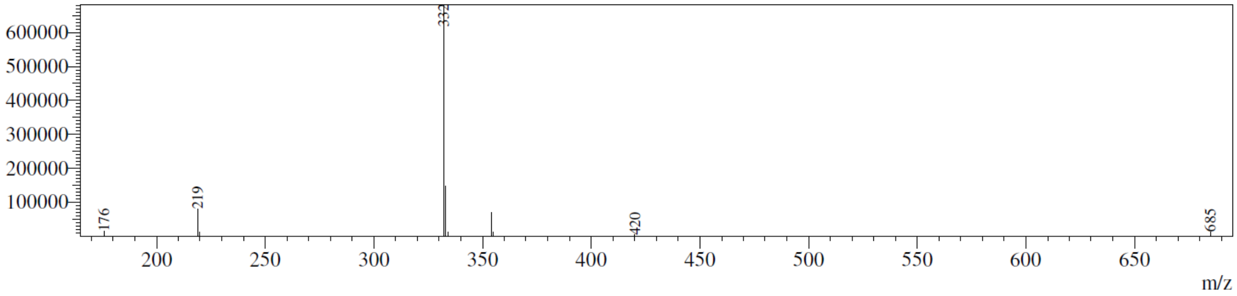

2710-33

(MW343)

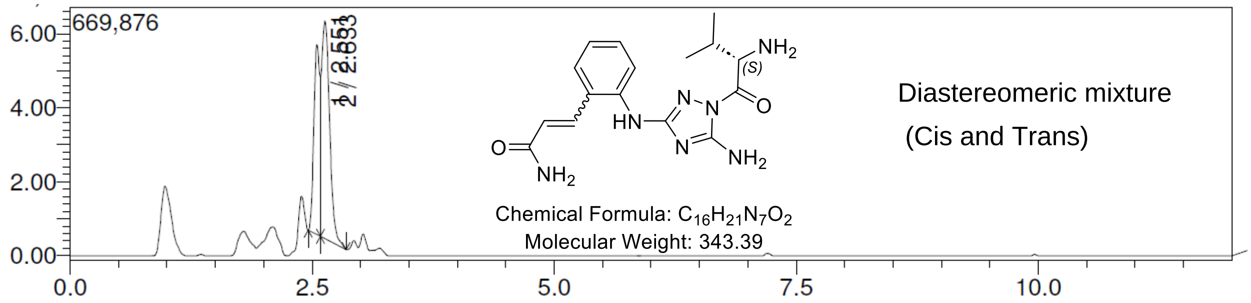

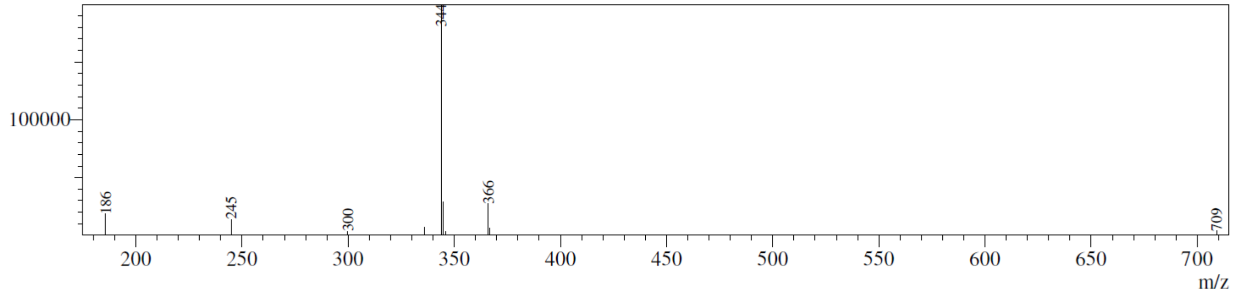

2710-34

(MW357)

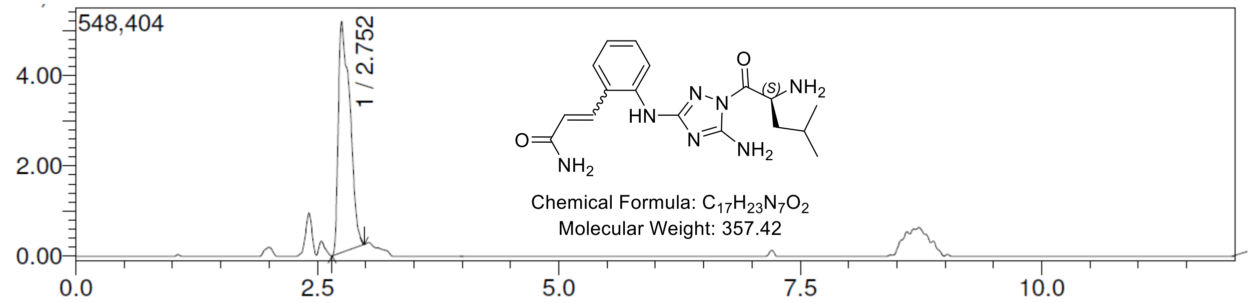

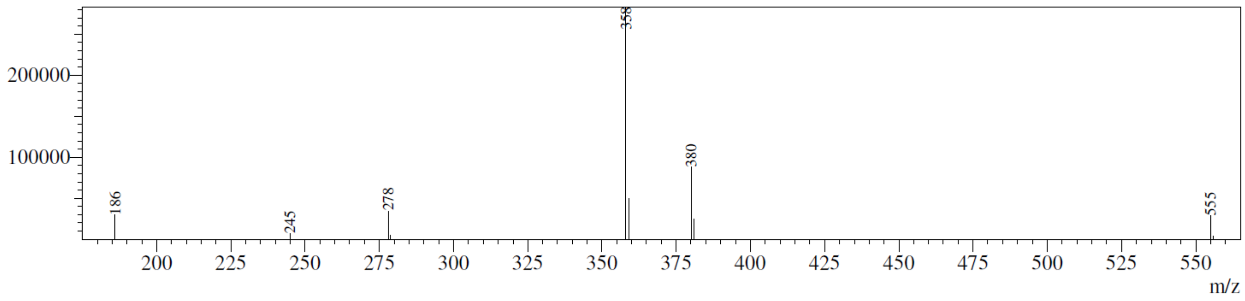

2710-33-467

(MW467)

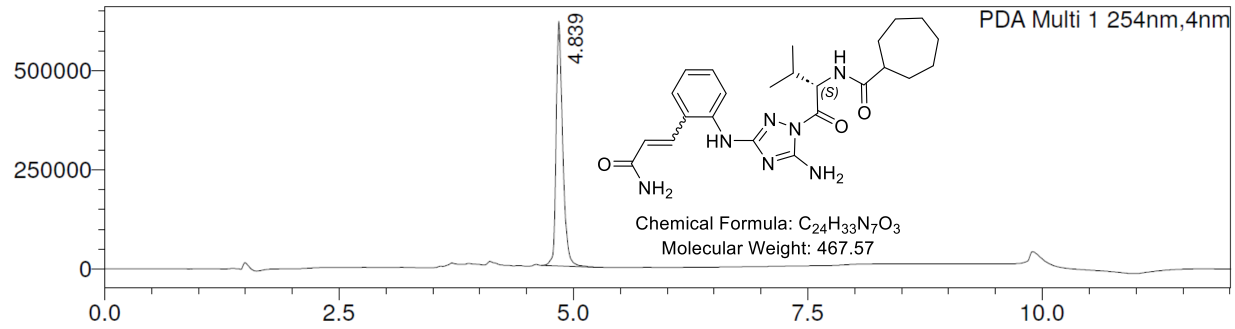

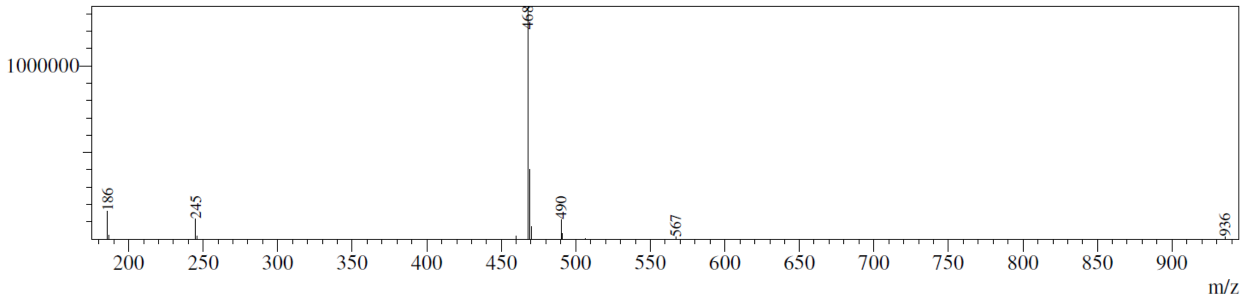

2710-34-481

(MW481)

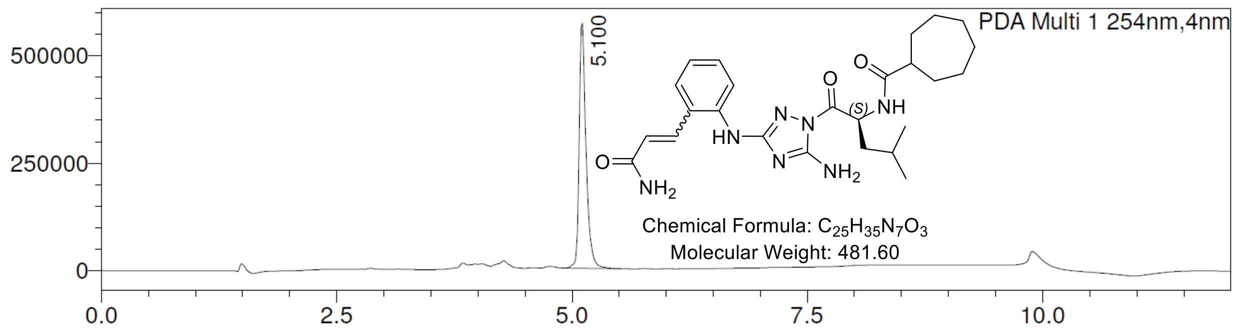

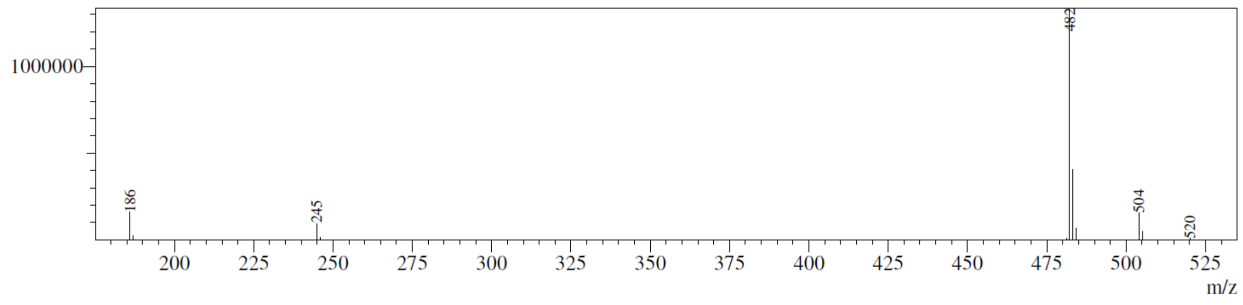

HRMS data of active compounds:

**2710-31: MH+:332.1959, [M+Na]: 354.1799**
**2710-33-343: MH+:344.2008, [M+Na]: 366.1854**
**2710-33-467: [M+Na]: 490.2842**
**2710-34-357: MH+:358.2187, [M+Na]: 380.2025**
**2710-34-481: MH+:482.3159, [M+Na]: 504.2998**

NMR data of 2-acetamido-3-(4-((5-amino-1-valyl-1H-1,2,4-triazol-3-yl) amino)phenyl)propenamide (2710-19):

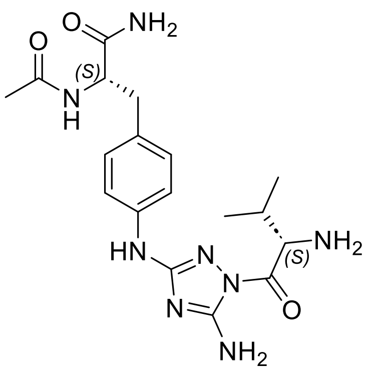

2710-19

^{1}H NMR (400 MHz, DMSO) δ 8.82 (s, 1H), 8.38 (s, 2H), 8.20 (s, 2H), 8.00- 7.93 (m, 1H) 7.81 (s, 1H), 7.39 (d, J = 8.0 Hz, 2H), 6.55 (s, 2H), 7.06 (d, J = 8.0 Hz, 2H), 3.80- 3.74 (m, 1H), 2.90 (dd, J = 16.0, 4 Hz, 1H), 2.68–2.60 (m, 2H), 2.17–2.14 (m, 1H), 1.77 (s, 3H), 0.98 (m, 6H): **^{13}C NMR (100 MHz, DMSO)** δ 173.89, 173.40, 170.1, 169.42, 129.67, 116.82, 116.16, 58.90, 54.55, 54.58, 37.50, 29.53, 23.01, 19.22, 19.22, 18.45, 17.39.

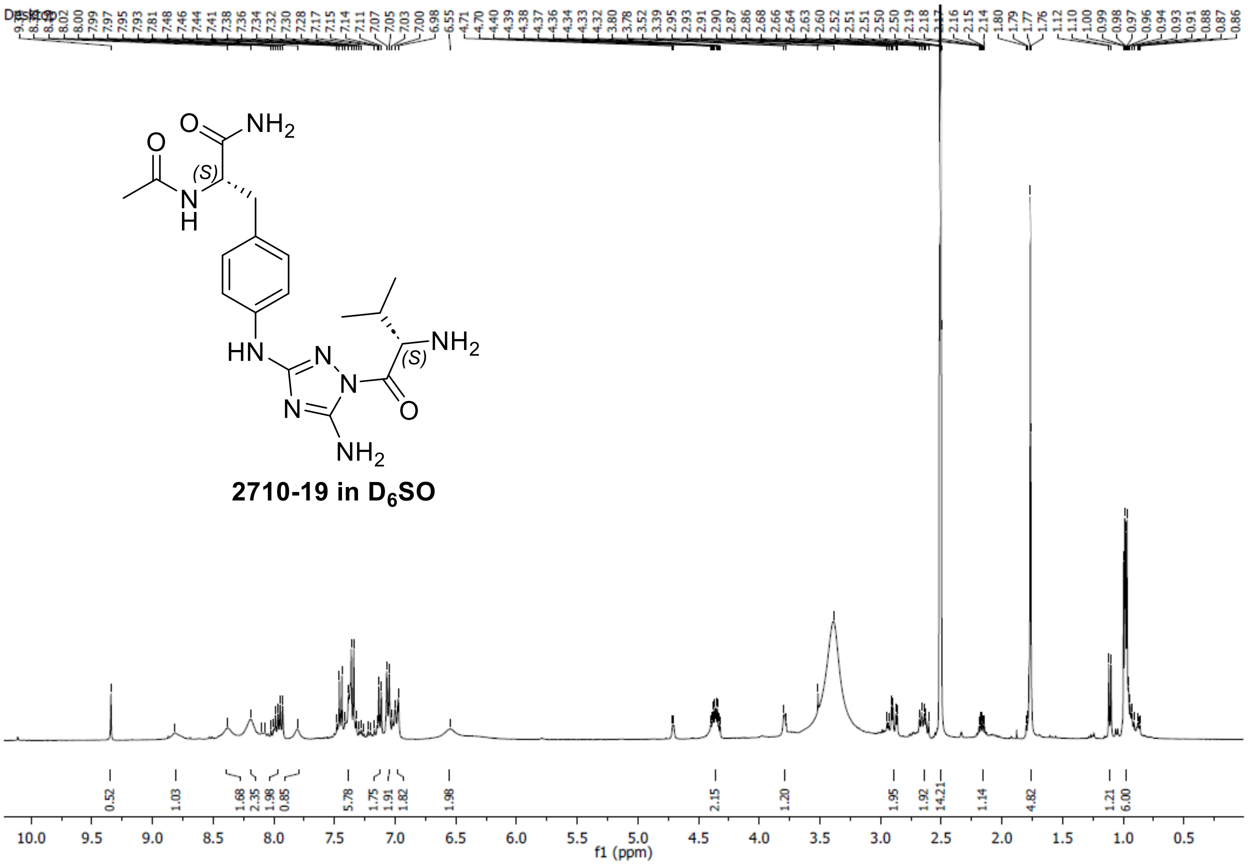
Desktop
O
NH2
(S)
N
H
HN
N
N
N
NH2
(S)
O
NH2
2710-19 in D6SO
f1 (ppm)

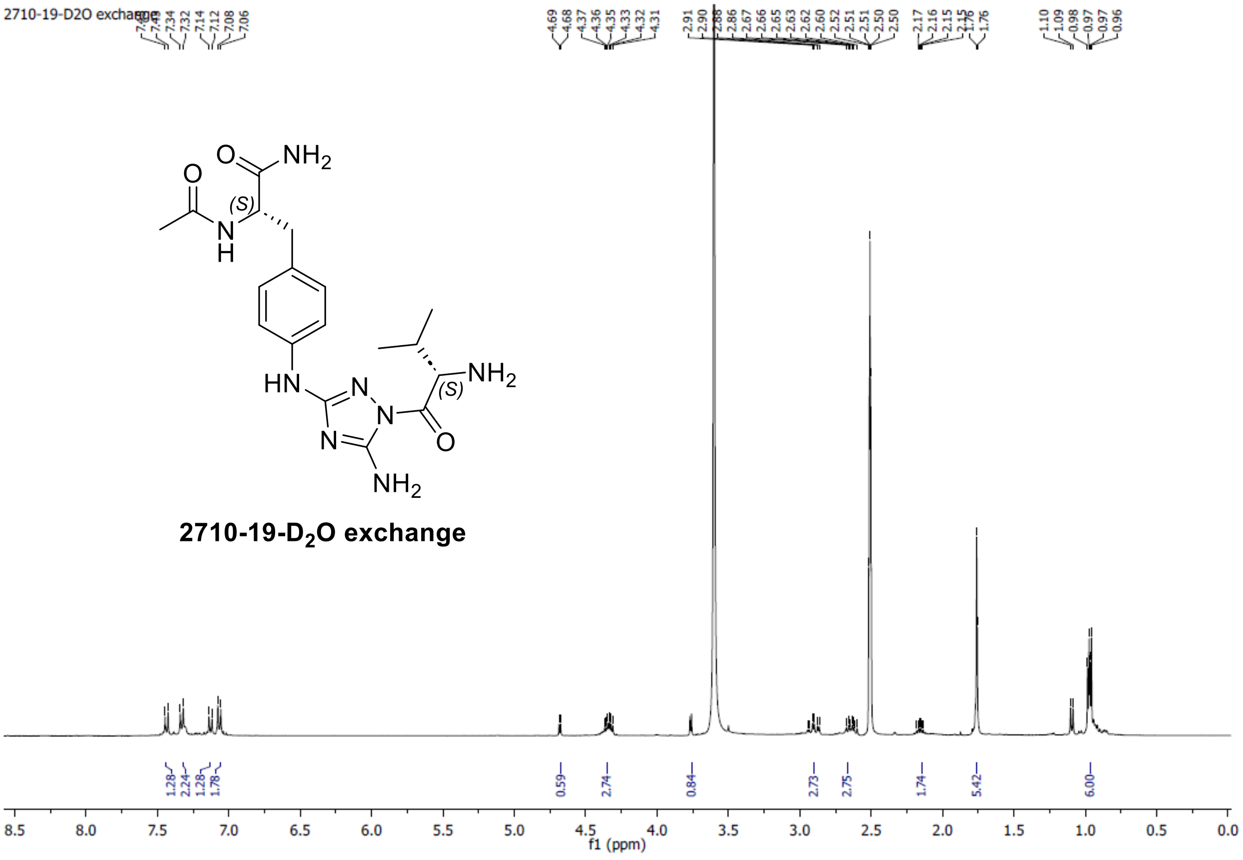
2710-19-D2O exchange
O
NH2
(S)
N
H
HN
N
N
N
NH2
(S)
O
NH2
2710-19-D2O exchange
f1 (ppm)

2710-19-D2O13C

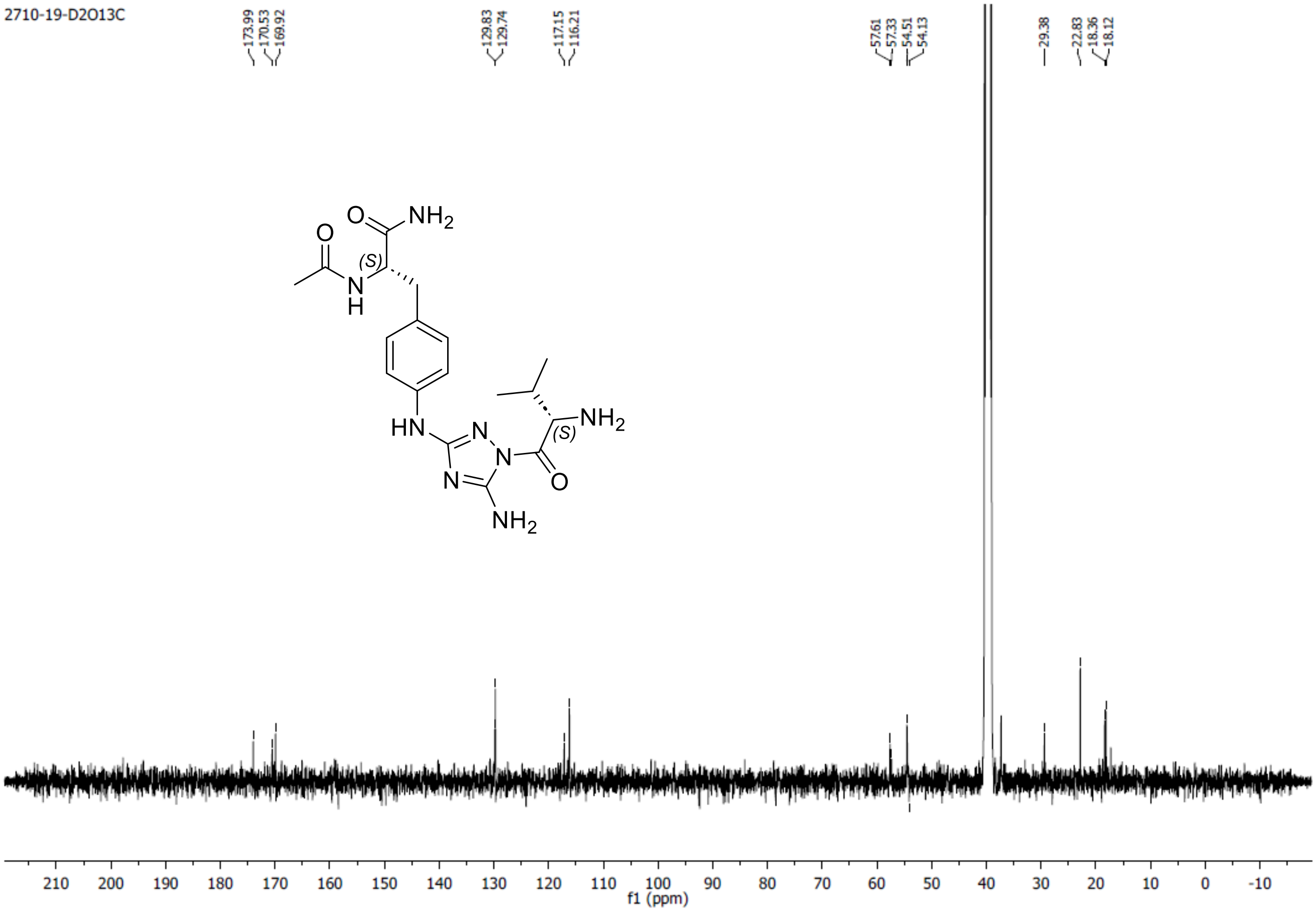

Supplement: Supplementary file 1 [file ijms-26-06193-s001.zip › ijms-3687658-supplementary.pdf]
